# Prevalence of Depressive Symptoms in Patients Undergoing Aseptic Revision Total Hip Arthroplasty Differs Based on Mode of Failure

**DOI:** 10.1016/j.artd.2025.101627

**Published:** 2025-02-13

**Authors:** Kristen I. Barton, Daniel N. Bracey, Vishal Hegde, Aviva Pollet, Roseann Johnson, Douglas A. Dennis, Jason M. Jennings

**Affiliations:** a Orthopaedic Surgery, Western University, London, Ontario, Canada; b Department of Orthopaedic Surgery, University of North Carolina School of Medicine Orthopaedics, Chapel Hill, NC, USA; c Department of Orthopaedic Surgery, Johns Hopkins University, Baltimore, MD, USA; d Orthopedic Surgery, Colorado Joint Replacement, Denver, CO, USA; e Department of Mechanical and Materials Engineering, University of Denver, Denver, CO, USA; f Department of Orthopaedics, University of Colorado School of Medicine, Denver, CO, USA; g Department of Biomedical Engineering, University of Tennessee, Knoxville, TN, USA

**Keywords:** Revision, Total hip arthroplasty, Depression, Depressive symptoms, Total hip replacement, Mental health

## Abstract

**Background:**

Complications following total hip arthroplasty (THA) may necessitate a revision and patients who go on to a revision THA may experience depressive symptoms. The objective of this study was to investigate the prevalence of depressive symptoms before and after revision THA for six different failure modes.

**Methods:**

Patients who underwent a THA revision with minimum 1-year follow-up at a single institution from 2008 to 2022 were retrospectively reviewed. Patients were grouped by failure modes: aseptic loosening, impingement, infection, instability, metallosis, polyethylene wear, and femoral stem pain. Preoperative and postoperative Veterans RAND 12-Item Health Scores and Harris Hip Score were compared.

**Results:**

Twenty-four percent of patients in the retrospective cohort review had a previous existing mental health diagnosis, with depression being the most common (18% of all patients). The prevalence of depressive symptoms differed significantly by failure mode both preoperatively (*P* = .002) and postoperatively (*P* = .019). Veterans RAND 12 mental component score was significant between mode of failure groups both preoperatively (*P* < .001) and postoperatively (*P* = .005). Function significantly improved in all groups from preoperatively to postoperatively. Patients with depressive symptoms had significantly lower physical component score with instability, aseptic loosening, stem pain, and metallosis preoperatively (*P* < .001) and with instability, aseptic loosening, stem pain, and polyethylene wear postoperatively (*P* = .002).

**Conclusions:**

Nearly 25% of patients with failed THA had a pre-existing mental health diagnosis and depressive symptoms were the most common. Unfortunately, depressive symptoms only improve modestly with revision surgery and can adversely affect a patient’s functional outcome.

## Introduction

Total hip arthroplasty (THA) is an appropriate treatment option for severe hip osteoarthritis and is one of the most reliable and reproducible procedures in orthopaedic surgery, achieving as high as a 97% success rate [1]. However, complications following primary THA may necessitate a revision. The most common indications for revision THA (rTHA) include instability/dislocation, mechanical loosening, impingement, metallosis, polyethylene wear, and infection. The need for an rTHA likely causes considerable anxiety and symptoms of depression. Patients with psychological disorders and depression undergoing lower extremity joint arthroplasty have been shown to have poorer postoperative outcomes, higher risk of infection, higher rates of dissatisfaction, increased readmission rates, and increased healthcare utilization [[2], [3], [4], [5], [6]]. Moreover, mental health disorders are a well-known risk factor for poor outcomes in both primary THA and rTHA [3].

In a previous systematic review, there was strong evidence that lower preoperative mental health (measures with the 12-item short form survey or 36-item short form survey) was associated with lower scores on function and pain after total knee arthroplasty (TKA). However, for THA, only limited, conflicting, or no evidence was found [7]. In another study in primary THA, low depression and anxiety levels were positively related to early functionality after THA [8]. Recently, authors demonstrated that depression and/or anxiety disorders are common in both primary THA (prevalence of 30%) and rTHA (prevalence of 33%) [2]. Furthermore, the authors demonstrated that patients with preoperative depressive and/or anxiety disorders were significantly less likely to report “much better” joint function after primary THA compared with those without these disorders at 2 years postoperatively.

There is minimal research that is known about mental health disorders and their association with failure in the setting of rTHA. Therefore, the primary objective of this study is to identify the prevalence of depressive symptoms both preoperatively and postoperatively in patients undergoing rTHA. Furthermore, our secondary objective was to determine if mode of failure was associated with prevalence of depressive symptoms and postoperative functional outcomes.

## Material and methods

### Study participants

Ethics approval was received from the Institutional Review Board at our institution (1607342). A retrospective cohort review of prospectively collected data for an observational cohort of all patients undergoing rTHA at a single institution by 6 fellowship-trained arthroplasty surgeons between September 1, 2008 and December 1, 2022 was conducted. Data were extracted from an electronic institutional database (OrTech). All patients who were followed for a 1-year period were included in the analysis. Indication for rTHA included the following: aseptic loosening, impingement, infection, instability, metallosis, polyethylene wear, or stem pain. Patients undergoing revision of the acetabular, femoral, or all components were included; however, those undergoing isolated liner exchange were excluded from the study. Patient demographics including age, sex, body mass index (BMI), and surgery type were extracted.

### Outcome measures

Patient-reported outcome measures including the Veterans RAND-12 Item Health Score, including both the mental component score (MCS) and physical component score (PCS), and Harris Hip Score (HHS) were extracted. All patient-reported outcome measures were evaluated preoperatively and at the most recent clinic follow-up appointment. Veterans RAND-12 MCS of < 42 was considered at risk for depressive symptoms [[9], [10], [11]]. Furthermore, each patient was also evaluated for an existing mental health diagnosis (anxiety, bipolar disorder with or without mania, depression, panic attacks, post-traumatic stress disorder, mood disorder, and obsessive-compulsive disorder) and number of medications at the time of revision surgery. The mental health diagnoses were established by our internal medicine perioperative team prior to the revision surgery. All patients underwent screening prior to surgical intervention.

### Statistical analysis

Descriptive statistics were used to summarize demographic data (age, sex, BMI, surgery type, and follow-up). One-way analysis of variances was used to assess MCS and PCS preoperatively and at the most recent follow-up, change in MCS and PCS over time, change in HHS over time, number of medications, and reason for revision. Tukey pairwise comparisons were conducted to determine the differences between means. Chi-square tests were used to examine differences between the presence of mental health disorders (< 42 on the MCS) and reason for revision. Fisher’s exact tests were used to determine if there were associations between depression preoperatively and reason for revision. Statistical analysis was conducted using Minitab Version 18 software (State College, PA) and significance for all statistical tests was accepted at *P* ≤ .05.

## Results

### Patient demographics

A retrospective cohort review was conducted and n = 410 rTHA patients were included. The reason for the rTHA surgery was the following: aseptic loosening (n = 111), impingent (n = 18), infection (n = 29), instability (n = 63), metallosis (n = 104), polyethylene wear (n = 76), and stem pain (n = 9) (Table 1). There were no differences between age (*P* = .296) and BMI (*P* = .104) for the reason for revision (Table 1). The mean age was 65.2 ± 11.8 years and the mean BMI was 27.5 ± 5.3 kg/m^2^. Follow-up was significantly different (*P* < .001) between groups for aseptic loosening (3.6 ± 2.5), impingent (4.3 ± 2.8), infection (2.1 ± 1.6), metallosis (2.7 ± 1.9), and polyethylene wear (4.0 ± 2.7) (Table 1). Sex was significantly different (*P* = .037) between groups for all modes of failure: aseptic loosening (male [M]: 36.9%; female [F]: 63.1%), impingement (M: 22.2%; F: 77.8%), infection (M: 89.7%; F: 10.3%), instability (M: 57.1%; F: 42.9%), metallosis (M: 41.3%; F: 58.7%), polyethylene wear (M: 48.7%; F: 51.3%), and stem pain (M: 33.3%; F: 66.7%) (Table 1). Existing mental health diagnoses in the patient population were the following: anxiety (13%), bipolar disorder (1%), depression (18%), panic attacks (1%), post-traumatic stress disorder (0.5%), a mood disorder (0.2%), or obsessive-compulsive disorder (0.2%) (Table 2). There was n = 312 (76%) patients with no prior mental health diagnoses. The number of medications was significantly different (*P* = .012) among revision mode for instability (0.54 ± 1.0), aseptic loosening (0.50 ± 0.8), and polyethylene wear (0.12 ± 0.4).

**Table 1:** Patient demographics.

Demographic	Aseptic loosening, n = 111	Impingement, n = 18	Infection, n = 29	Instability, n = 63	Metallosis, n = 104	Poly wear, n = 76	Stem pain, n = 9	*P* value
Age (years)	65.2 ± 11.8	60.0 ± 12.9	63.5 ± 10.9	66.6 ± 10.5	66.1 ± 9.5	65.2 ± 11.3	62.1 ± 11.1	.296
BMI (kg/m^2^)	27.5 ± 5.3	24.9 ± 4.6	30.0 ± 6.0	28.0 ± 5.8	29.3 ± 13.6	26.5 ± 4.7	30.4 ± 5.3	.104
Follow-up (years)	3.6 ± 2.5	4.3 ± 2.8	2.1 ± 1.6	3.1 ± 2.4	2.7 ± 1.9	4.0 ± 2.7	4.3 ± 3.2	<.001
Sex	M (41): 36.9% F (70): 63.1%	M (4): 22.2% F (14): 77.8%	M (26): 89.7% F (3): 10.3%	M (36): 57.1% F (27): 42.9%	M (43): 41.3% F (61): 58.7%	M (37): 48.7% F (39): 51.3%	M (3): 33.3% F (6): 66.7%	.027

BMI, body mass index; F, female; M, male.

**Table 2:** Existing mental health diagnosis in patient population.

Mode of failure	Anxiety	Bipolar disorder	Depression	Panic attacks	Post-traumatic stress disorder	Mood disorder	Obsessive-compulsive disorder	None
Instability	10	1	14	1	1			45
Infection	5		6	1				22
Impingement	2		4					13
Aseptic loosening	20	3	25	1	1	1		76
Metallosis	12	1	20	1			1	77
Poly wear	2		2					73
Stem pain	3		3					6

#### Patient-reported outcomes

HHS was significantly different for both preoperatively (*P* < .001) and postoperatively (*P* = .003) in mode of failure (Table 3). Preoperatively, polyethylene wear (77.6 ± 18.5), metallosis (67.1 ± 21.4), and instability (65.3 ± 21.9) had the highest HHS for the reason for failure. Postoperatively, polyethylene wear (84.1 ± 17.4), metallosis (79.2 ± 17.9), and stem pain (74.7 ± 14.8) had the highest HHS for the reason for failure. All failure modes increased HHS postoperatively (Table 3).

**Table 3:** Analysis of preoperative and postoperative HHS, function, and range of motion for each reason for failure.

Outcome	Aseptic loosening, n = 111	Impingement, n = 18	Infection, n = 29	Instability, n = 63	Metallosis, n = 104	Poly wear, n = 76	Stem pain, n = 9	*P* value
Total HHS preoperatively	49.6 ± 19.8	51.9 ± 13.3	50.6 ± 19.8	65.3 ± 21.9	67.1 ± 21.4	77.6 ± 18.5	53.9 ± 26.1	<.001
Total HHS postoperatively	74.4 ± 18.9	67.1 ± 19.2	71.6 ± 25.1	73.7 ± 23.0	79.2 ± 17.9	84.1 ± 17.4	74.7 ± 14.8	.003
Function preoperatively	24.7 ± 12.1	29.7 ± 10.3	22.7 ± 10.3	31.4 ± 11.0	34.5 ± 9.8	38.2 ± 9.8	27.6 ± 12.9	<.001
Function postoperatively	32.9 ± 11.6	33.7 ± 8.8	32.6 ± 12.6	36.6 ± 8.5	36.6 ± 8.5	38.6 ± 9.3	36.6 ± 8.1	.003
ROM preoperatively	92.1 ± 17.9	97.8 ± 14.2	89.2 ± 6.9	90.8 ± 35.2	97.7 ± 12.6	97.1 ± 28.2	99.6 ± 11.3	.247
ROM postoperatively	99.3 ± 11.0	100.3 ± 12.8	95.2 ± 12.1	103.3 ± 11.9	101.0 ± 11.1	102.9 ± 13.1	100.0 ± 8.2	.056

HHS, Harris Hip Score; ROM, range of motion.

MCS improved in all reason for failure groups postoperatively (Table 4). MCS was significant between mode of failure groups both preoperatively (*P* < .001) and postoperatively (*P* = .005). Preoperatively, polyethylene wear (55.5 ± 9.3), metallosis (50.4 ± 12.0), and instability (50.7 ± 12.5) had the highest MCS preoperatively (Table 4). Also, polyethylene wear (57.3 ± 7.9), metallosis (54.0 ± 10.7), and instability (51.5 ± 12.6) had the highest MCS postoperatively (Table 4). Interestingly, stem pain and impingement had the lowest scores preoperatively (40.7 ± 15.7 and 44.1 ± 15.9, respectively) and postoperatively (50.4 ± 14.6 and 46.8 ± 12.3, respectively). PCS was significant between mode of failure groups both preoperatively (*P* < .001) and postoperatively (*P* = .002). Of note, the PCS improved over time for the following failure groups: aseptic loosening, impingement, infection, instability, and metallosis (Table 4).

**Table 4:** Analysis of MCS and PCS preoperatively and postoperatively.

Outcome	Aseptic loosening, n = 111	Impingement, n = 18	Infection, n = 29	Instability, n = 63	Metallosis, n = 104	Poly wear, n = 76	Stem pain, n = 9	*P* value
VR-12 MCS preoperatively	49.3 ± 13.3	44.1 ± 15.9	46.9 ± 9.7	50.7 ± 12.5	50.4 ± 12.0	55.5 ± 9.3	40.7 ± 15.7	< .001
VR-12 MCS postoperatively	53.0 ± 12.3	46.8 ± 12.3	51.6 ± 12.5	51.5 ± 12.6	54.0 ± 10.7	57.3 ± 7.9	50.4 ± 14.6	.005
VR-12 PCS preoperatively	30.7 ± 8.9	33.5 ± 9.2	30.6 ± 10.2	34.1 ± 10.5	37.9 ± 11.6	44.2 ± 11.2	34.4 ± 9.6	< .001
VR-12 PCS postoperatively	38.5 ± 10.6	35.7 ± 13.1	39.1 ± 11.0	36.7 ± 12.7	41.0 ± 11.5	44.0 ± 12.0	34.4 ± 12.7	.002

MCS, mental component score; PCS, physical component score; VR-12, Veterans RAND-12.

When assessing MCS < 42 (positive screen for depression), there was a reduction in the percentage of MCS < 42 in the following groups postoperatively (Table 5): aseptic loosening (27.3%-20.7%), impingement (50.5% to 38.9%), infection (31.0% to 27.6%), instability (25.8%-22.2%), metallosis (26.0%-13.5%), and stem pain (44.4%-22.2%). However, there was no improvement in MCS in the polyethylene wear group (7.9% both preoperatively and postoperatively). MCS < 42 was significantly different for both preoperatively (*P* = .002) and postoperatively (*P* = .019) in mode of failure (Table 5) between reason for failure. When evaluating the percentage of patients with MCS < 42 within each mode of failure, the only group that had a significant change between their preoperative and postoperative visits was the metallosis group (*P* = .036) (Table 5).

**Table 5:** Analysis of the change in percentage of patients with an MCS < 42 and the reason for revision over time.

Surgical timeframe	Aseptic loosening, n = 111	Impingement, n = 18	Infection, n = 29	Instability, n = 63	Metallosis, n = 104	Poly wear, n = 76	Stem pain, n = 9
Preoperatively	27.3% (n = 30)	50.0% (n = 9)	31.0% (n = 9)	25.8% (n = 16)	26.0% (n = 27)	7.9% (n = 6)	44.4% (n = 4)
Postoperatively	20.7% (n = 23)	38.9% (n = 7)	27.6% (n = 8)	22.2% (n = 14)	13.5% (n = 14)	7.9% (n = 6)	22.2% (n = 2)
*P* value	.273	.738	1.000	.680	.036	1.000	.620

MCS, mental component score.

#### Functional outcomes

Function was significantly different between reason for revision group both preoperatively (*P* < .001) and postoperatively (*P* = .003) (Table 3). Range of motion was not significantly different between reason for revision group either preoperatively (*P* = .247) or postoperatively (*P* = .056) (Table 3).

## Discussion

The present study investigated the prevalence of depressive symptoms both preoperatively and postoperatively in patients undergoing rTHA. In our retrospective cohort review, we found that 24% of patients had a previous existing mental health diagnosis, with depression being the most common. Furthermore, the prevalence of depressive symptoms differed significantly by failure mode both preoperatively and postoperatively. When assessing those with depressive symptoms, there was improvement in the MCS in the following groups postoperatively: aseptic loosening, impingement, infection, instability, metallosis, and stem pain. Unfortunately, depressive symptoms only improved modestly with revision surgery and can adversely affect a patient’s functional outcome. Finally, function significantly improved in all groups from preoperatively to postoperatively, yet range of motion did not significantly improve within groups. Patients with depressive symptoms had significantly lower PCS both preoperatively and postoperatively in failure for instability, aseptic loosening, and stem pain.

Singh and Lewallen [12] have previously found that depression, which is considered a modifiable clinical factor, was an important independent predictor of pain, functional limitation, and use of pain medications, following rTHA. Not surprisingly, the authors also found that depression was associated with higher risk of activity limitation at 5 years after rTHA [12]. Another study [13] investigating the relationship of depression and revision arthroplasty demonstrated by a multivariate analysis that preoperative depression was associated with extended length of stay, nonhome discharge, 90-day readmission, 90-day emergency department visit, prosthetic joint infection, revision surgery, and increased costs. Also, they found that preoperative depression was present in 13.1% of rTHA patients and 14.4% of revision TKA (rTKA) patients [13]. Also, in rTKA, the authors found that depression was associated with extended length of stay, nonhome discharge, 90-day readmission, 90-day emergency department visit, revision surgery, and increased costs [13]. Similar to the present study, a diagnosis of depression was highly prevalent in our rTHA population preoperatively (18%). In a previous study from our research group [14], nearly a quarter of all aseptic rTKA patients met criteria for depressive symptoms based on an MCS less than 42 at the time of revision surgery as well. In their population of rTKA, this then decreased to 18.8% at the latest follow-up visit postoperatively. However, the authors concluded that the prevalence of depressive symptoms in rTKA patients was independent of failure mode [14], which was not what we found in the rTHA population as the prevalence of depressive symptoms differed significantly by failure mode both preoperatively and postoperatively.

The presence of depressive or anxiety disorders prior to both primary and revision THA and TKA is common, and associated with a significantly higher risk of infection, revision, reoperation, and patient dissatisfaction [2]. Interestingly, Harmer *et al.* [2] has demonstrated that patients with preoperative depressive and/or anxiety disorders were significantly less likely to report “much better” joint function even after primary THA or TKA, compared with those without these disorders at 2 years postoperatively. In a previous study, Hedge *et al.* [15] demonstrated that patients undergoing 2-stage exchange for periprosthetic joint infection (PJI) have a 4 times higher prevalence of preoperative depressive symptoms than when they were undergoing an aseptic revision. Also, patients undergoing debridement with implant retention with depression or preoperative depressive symptoms have lower functional scores postoperatively [15]. Furthermore, a previous study has shown that the risk of depressive and stress disorders was significantly higher in those who underwent PJI-THA when compared to aseptic revision, with the added risk of bipolar when compared to primary THA [16]. Patients who undergo spacer placement for septic rTHA or rTKA have a disproportionately higher incidence of mental health disorders within 2 years following surgery when compared to those undergoing aseptic revisions and primary procedures [16]. In addition, Furdock *et al.* [17] demonstrated that 6.4% of aseptic revision patients (both THA and TKA) developed Patient-Reported Outcomes Measurement Information System depression scores consistent with major depressive disorder during treatment, compared to 20% of 2-stage revision arthroplasty patients for septic revision. Although this study investigated aseptic rTHA, orthopaedic surgeon screening of PJI patients with referral for treatment of depression may help improve outcomes postoperatively [15] and surgeons should strongly consider collaborative care with psychiatrists or mental health professionals [16]. Furthermore, mental health optimization to be of similar importance to preoperative variables such as diabetic control, prior to arthroplasty [2].

This study is not without limitations. First, the study is a retrospective review and there is potential for both selection and/or recall biases. Second, there are different classifications of depression and mental health disorders within the *Diagnostic and Statistical Manual of Mental Disorders Fifth Edition*. As previously described [[9], [10], [11]], MCS < 42 has been previously used as a standardized method to determine depressive symptoms which the rationale for using this method in the present study. However, note the authors recognize that this may not capture all mental health disorders within the study due to the *Diagnostic and Statistical Manual of Mental Disorders Fifth Edition* criteria as medical records were screened for diagnoses. Furthermore, medical records were also used to determine the medications each patient was prescribed at the time of revision surgery. The authors were also not able to identify which patients may have been receiving treatment for their mental health disorder, which may influence reported outcomes. Finally, the number of patients within each failure mode group may be underpowered to detect statistically significant differences for specific outcomes in this study.

## Conclusions

In summary, nearly 25% of patients with failed THA had a pre-existing mental health diagnosis and depressive symptoms were the most common. Unfortunately, depressive symptoms only improve modestly with revision surgery and can adversely affect a patient’s functional outcome. Therefore, surgeon awareness of failure modes predisposed to depressive symptoms may improve diagnosis and treatment of depression and potentially improve patient expectations and outcomes after rTHA.

## Conflicts of interest

Jason M. Jennings received royalties from Total Joint Orthopedics; is a paid consultant for Total Joint Orthopedics and Xenex; holds stock or stock options in Xenex; received research support from DePuy, a Johnson & Johnson Company as a Principal Investigator; and is a board member of AAOS and American Association of Hip and Knee Surgeons. Douglas A. Dennis received royalties from Corin USA, DePuy, a Johnson & Johnson Company, and Innomed; received speakers bureau/paid presentations for Corin USA and DePuy, a Johnson & Johnson Company; is a paid consultant for Corin USA and DePuy, a Johnson & Johnson Company; holds stock or stock options in Corin USA and Joint Vue; received research support from DePuy, a Johnson & Johnson Company and AdventHealth Porter as a Principal Investigator; received royalties, financial or material support from Wolters Kluwer Health - Lippincott Williams & Wilkins; and is in the medical/orthopaedic publications editorial/governing board of Orthopedics Today. Vishal Hegde is a paid consultant for Globus Medical; received research support from Ortho Development as a Principal Investigator; received other financial or material support from Smith & Nephew; and is a board member of the American Association of Hip and Knee Surgeons. All other authors declare no potential conflicts of interest.

For full disclosure statements refer to https://doi.org/10.1016/j.artd.2025.101627.

## CRediT authorship contribution statement

**Kristen I. Barton:** Writing – review & editing, Writing – original draft, Investigation, Formal analysis. **Daniel N. Bracey:** Writing – review & editing, Investigation, Conceptualization. **Vishal Hegde:** Writing – review & editing, Project administration, Conceptualization. **Aviva Pollet:** Writing – review & editing, Project administration, Data curation. **Roseann Johnson:** Writing – review & editing, Project administration, Methodology, Data curation, Conceptualization. **Douglas A. Dennis:** Writing – review & editing, Supervision, Methodology, Investigation, Conceptualization. **Jason M. Jennings:** Writing – review & editing, Supervision, Methodology, Investigation, Data curation, Conceptualization.

## Appendix A Supplementary data

Conflict of Interest Statement for Pollet

Conflict of Interest Statement for Dennis

Conflict of Interest Statement for Bracey

Conflict of Interest Statement for Jennings

Conflict of Interest Statement for Barton

Conflict of Interest Statement for Johnson

Conflict of Interest Statement for Hegde
