# Supplementary material for: Prevalence of Depressive Symptoms in Patients Undergoing Aseptic Revision Total Hip Arthroplasty Differs Based on Mode of Failure
Source: Arthroplast Today. 2025 Feb 13;32:101627. doi: 10.1016/j.artd.2025.101627 (PMC11874526; doi:10.1016/j.artd.2025.101627)
Supplement: Conflict of Interest Statement for Barton [file mmc5.doc]

# CONFLICT OF INTEREST STATEMENT

***The Journal of Arthroplasty***

(Adopted from the American Academy of Orthopaedic Surgeons disclosure statement)

The following form **must be filled out completely and submitted by each author (example, 6 authors, 6 forms). If no discloser is required, please write/type “none” at the end of each sentence.**

Manuscript Title: **Prevalence of Depressive Symptoms in Patients Undergoing Aseptic Revision Total Hip Arthroplasty Differs Based on Mode of Failure**

1. Royalties from a company or supplier (The following conflicts were disclosed)

None

2. Speakers bureau/paid presentations for a company or supplier (The following conflicts were disclosed)

None

3A. Paid employee for a company or supplier (The following conflicts were disclosed)

None

3B. Paid consultant for a company or supplier (The following conflicts were disclosed)

None

3C. Unpaid consultants for a company or supplier (The following conflicts were disclosed)

None

4. Stock or stock options in a company or supplier (The following conflicts were disclosed)

None

5. Research support from a company or supplier as a Principal Investigator (The following conflicts were disclosed)

None

6. Other financial or material support from a company or supplier (The following conflicts were disclosed)

None

7. Royalties, financial or material support from publishers (The following conflicts were disclosed)

None

8. Medical/Orthopaedic publications editorial/governing board (The following conflicts were disclosed)

None

9. Board member/committee appointments for a society (The following conflicts were disclosed)

None

**Each author must sign AND print or type his/her name, date and submit a separate form**

In addition, one BLINDED Conflict of Interest form (no author names used) should be submitted per manuscript with all author disclosures.

Kristen Barton Kristen Barton 7/25/24

Author Name (Print or Type) Author Signature Date
